# Cortactin in cancer cell migration and invasion

**DOI:** 10.18632/oncotarget.21088

**Published:** 2017-09-19

**Authors:** Miao Yin, Wenqing Ma, Liguo An

**Affiliations:** ^1^ Shandong Provincial Key Laboratory of Animal Resistance Biology, College of Life Sciences, Shandong Normal University, Jinan 250014, China

**Keywords:** cortactin, Arp2/3 complex, cancer cell, cell migration, cell invasion

## Abstract

Cortactin, a substrate of sarcoma (Src) kinases, is an actin-binding protein that is involved in cytoskeletal regulation, and is frequently overexpressed in cancer cells. Binding to the actin related protein 2/3 (Arp2/3) complex stimulates cortactin activity, which promotes F-actin nucleation and assembly. Cortactin promotes cancer cell migration and invasion, and plays a pivotal role in invadopodia formation and extra cellular matrix degradation. Overexpression of cortactin, by amplification of the chromosomal band 11q13, increases tumor aggressiveness. In this review, we report on the current knowledge and potential mechanisms of action of cortactin as a critical mediator of cancer cell migration and invasion.

## INTRODUCTION

Cortactin, a prominent actin-binding protein, was initially character as a component of the Src non-receptor tyrosine kinase pp60^src^. The name cortactin derives from the localization “cortical actin” structures [1, 2]. Cortactin acts as an actin assembly protein and plays a role in the assembly of branched actin and maintaining its stability via bind an Arp2/3 complex [3, 4]. Indeed, cortactin has been found at sites of dynamic actin assembly incellular protrusions, such as in lamellipodia and invadopodia [5, 6]. Cortactin can be phosphorylated by tyrosine and serine/threonine kinases [7], making cortactin an important regulatory target. Moreover, cortactin is critical for membrane trafficking and promotes the secretion of extracellular matrix (ECM)-degrading proteinases, which are crucial for the invadopodia formation and its function in tumor cell metastasis. Cortactin has been thus recognized for its association with cell motility and invasion [8, 9].

Cortactin has been demonstrated to be associated with cancer. For example, gene amplification studies have shown that the gene EMS1 (from human) encodes cortactin, which was overexpressed in a variety of tumors, such as breast cancers as well as head and neck tumors [10]. In addition, overexpression of cortactin enhances cell migration, invasion, and tumor cell metastasis [11].

Here, we provide an overview of the roles of cortactin, emphasizing the interaction with the actin cytoskeleton as well as the roles of cancer cell migration and invasion.

## CORTACTIN INTERACTS WITH ACTIN CYTOSKELETON

### Cortactin structure

**Figure 1:**
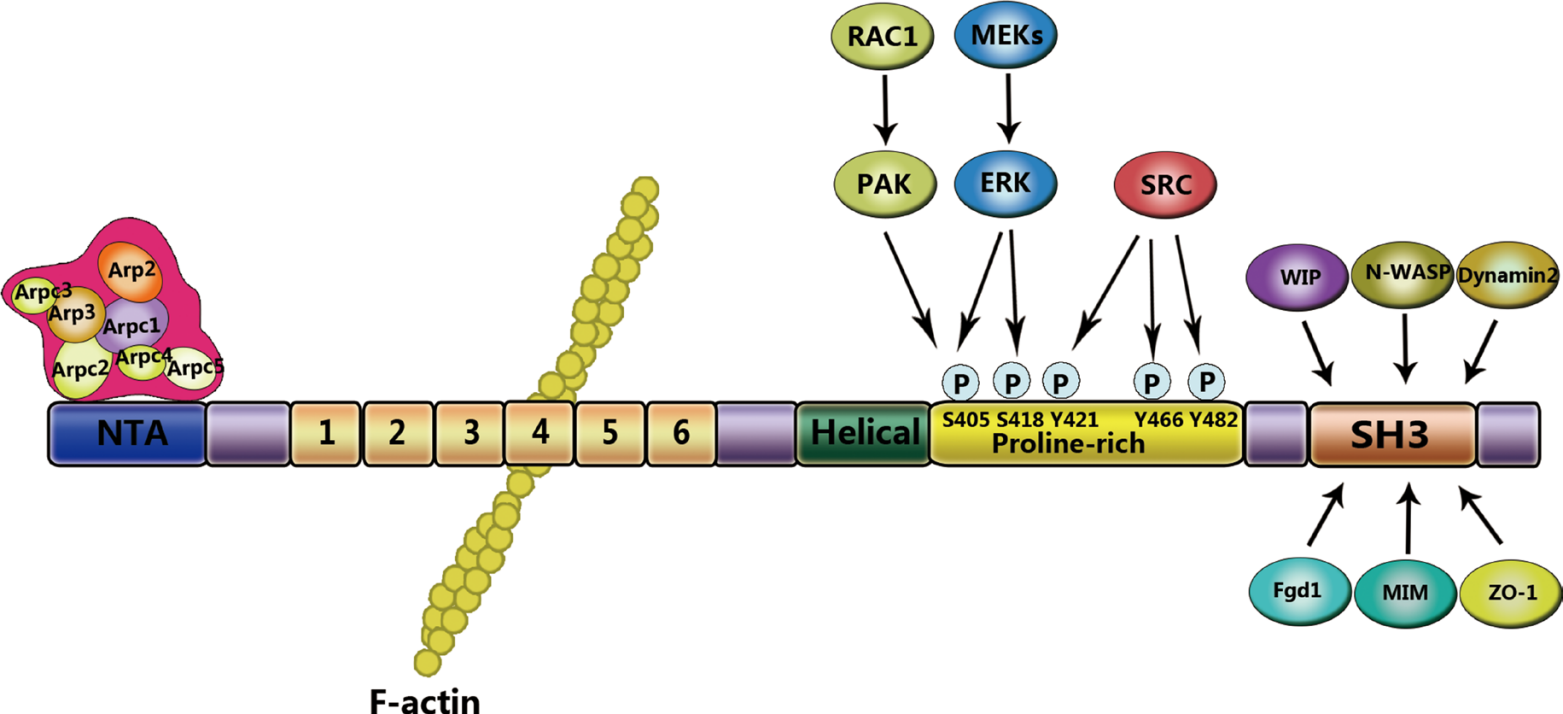
Structural domains of cortactin Cortactin consists of distinct domains that interact with different signaling molecules. The NTA domain is located at the N-terminus and interacts with and directly activates the Arp3 subunit of the Arp2/3 complex. The fourth repeat of the cortactin repeats region binds to F-actin. At the C-terminus, the proline-rich domain contains phosphorylation sites for serine/tyrosine kinases. The SH3 domain mediates binding to cytoskeletal, membrane trafficking, and signaling proteins.

Cortactin, a 63–65 kDa protein, was firstly identified as a phosphorylated substrate of v-Src-transformed chick embryo fibroblasts [12, 13]. The prediction of cortactin structure based on amino acid sequence shows that cortactin consists of four major domains: N-terminal acidic (NTA), 6.5 tandems repeat, proline-rich domain, and the C-terminal Src homology 3 domains (SH3 domains) [14, 15] (Figure 1). The NTA domain play a role in binding and activating the Arp2/3, and the process is involved in dynamic assembly of branched actin networks [16]. The NTA domain is localized between amino acid residues 15 and 35, and contains a conserved aspartic acid-aspartic acid-tryptophan (DDW) motif at residues 20–22, which is required for binding the Arp3, a subunit of Arp2/3 [17]. The DDW motif has highly conserved sequence, and its function is equivalent to the verprolin-cofilin-acidic (VCA) domain which is the Wiskott-Aldrich syndrome protein (WASP) family proteins [16, 17]. The NTA region is followed by a series of six complete 37 amino acid tandemly repeating segments, and one incomplete segment 20 residues in length [18]. These repeats, named ‘cortactin repeats’ [19], form a helix-turn-helix motif [12]. The cortactin repeats mediate cortactin binding to F-actin along with its co-localization with the cortical actin network [15, 16]. The fourth repeat is required to binding F-actin, whereas the third and fifth repeat are essential for maintaining the binding efficiently [20]. Derived from reversible polymerization of G-actin, F-actin supply the structural framework and mechanical forces that regulate morphological changes within the cell [20]. The process is also promoted by the Arp2/3 complex, which makes F-actin branching start [21, 22]. Following the 6.5 tandems repeat region, a proline-rich domain [23] is an α-helical domain of variable length (48–52 amino acid residues) that is enriched in sites of tyrosine, serine, threonine phosphorylation residues [12]. And a SH3 domain at the distal C-terminus mediates the interaction with proteins involved in diverse cellular functions. An abbreviated list of proteins that interact with cortactin is presented in Table 1. A great many of cytoskeletal proteins involved in the process of membrane trafficking, which bind to the cortactin SH3 domain, indicated that the cortactin acts as scaffold in the arrangement of cytoskeleton and the membrane trafficking [24].

Table 1Cortactin-binding partners

**Table:** (A) Binding partners of cortactin associated with actin assembly

Cortactin binding proteins	Cortactin binding site	Function
Arp2/3	NTA	Actin nucleation
Actin filaments	Repeat regions	Cytoskeletal polymer
Caldesmon	N-terminus	Actin binding protein
N-WASP	SH3	Actin assembly
WIP	SH3	Adaptor protein, Actin binding/assembly, WASP stabilization
MIM	SH3	Adaptor protein, Actin binding and regulation

**Table:** (B) Binding partners of cortactin associated with phosphorylation and acetylation

Cortactin binding proteins	Cortactin binding site	Function
HDAC6	Repeat region	Deacetylase
SIRT1	Repeat regions	Deacetylase
Met	Unknown	Receptor tyrosine kinase
Syk	Unknown	Tyrosine kinase
PTP1B	Tyr446	Tyrosine phosphatase
Nck1	Phospho-Y421, 466	Signaling adaptor
Src family kinases (Src, Fer)	phospho-Y421, 466, 482	Tyrosine kinase
ERK1/2	S405, 418	Serine/threonine kinase
PAK1	S113	Serine/threonine kinase
Abl/Arg	SH3	Tyrosine kinase

**Table:** (C) Binding partners of cortactin associated with membrane trafficking

Cortactin binding proteins	Cortactin binding site	Function
p120 catenin	N-terminus	Cell-cell adhesion via cadherin stability &trafficking
Hip1R	SH3	Membrane trafficking
ASAP1/AMAP1	SH3	Membrane trafficking, Signal transduction
Dynamin2	SH3	GTPase, Membrane trafficking
BK channels	SH3	Membrane excitability
K+ channel Kv1.2	Unknown	Membrane excitability

**Table:** (D) Binding partners of cortactin associated with signal transmission

Cortactin binding proteins	Cortactin binding site	Function
Grb2	N-terminus	Signaling adaptor
BPGAP1	SH3	RhoA-GAP
CortBP1/SHANK2	SH3	Synaptic plasticity, adaptor protein, regulates Na+/H+ exchanger 3
FGD1	SH3	Cdc42-GEF
ASAP1/AMAP1	SH3	Membrane trafficking, Signal transduction

### SH3 domain-binding partners

The principal function of cortactin is activating the actin cytoskeleton assembly via promoting and stabilizing the branched chained F-actin networks is induced by Arp2/3 complex [17]. The underlying mechanisms involved in this process have been described in several reviews [25, 26]. In addition, cortactin interacts with multiple binding partners at the site of actin polymerization.

The identification of cortactin SH3 domain-binding partners has provided novel insight into how cortactin promotes distinct cellular processes [27]. These proteins include Neural WASP (N-WASP), WASP-interacting protein (WIP), and dynamin 2 [28]. Among them N-WASP activates the Arp2/3 complex strongly [17, 29]. In spite of the fact that cortactin and N-WASP are able to binding the Arp2/3 and initiating the polymerization of actin [16, 17, 30], cortactin is weaker than N-WASP in stimulating the Arp2/3 [4, 16, 31]. Recent studies have shown that localization of cortactin was similar with N-WASP, as they are all localized at the site of actin assembly [17, 32]. Consequently, a pattern has been proposed where the Arp2/3 interacts with N-WASP and cortactin in sequence [17]. Although the main function of cortactin involves stabilizing of filament branches, N-WASP alone or in combination with cortactin stimulates the Arp2/3, bring forth the branched chained polymerization of F-actin [17, 30]. WIP also binds to the SH3 domain and promotes the capable of cortactin activate to Arp2/3 [13, 33]. Similarly, the cortactin-binding domain affects the localization of WIP at the cell cortex area [13, 34]. The association between cortactin-WIP complex and actin filaments, are essential for the maximal achievement of Arp2/3 activity, indicating a new finding that cortactin can link WIP to pre-existing filaments [13, 35]. WIP is also involved in the process of reducing the depolymerization of actin filaments and stabilizing the actin branch points [13, 36]. Dynamin 2 interacts with cortactin and this interaction, which is necessary for actin polymerization, and the SH3 domain and two motifs within the dynamin proline-rich domain serve as mediator in this process [17, 37].

### Cortactin activates the Arp2/3 complex and regulates the assembly of branched actin

The Arp2/3 complex is the main molecular regulator of actin polymerization, which is necessary for acting nucleation and forming a branched actin filament networks within cells [38, 39]. The branched actin networks provide to the structural of plasma membrane and processes, for example the formation of lamellipodia [40], pathogen motility, vesicle trafficking, and formation of cell–cell junctions [41, 42].

In vitro, half of the N-terminal portion of cortactin (amino acid residues 1–326) can be used to bind F-actin and induct the actin filament assembly via stimulating the Arp2/3 [16, 43]. Cortactin directly binds to Arp2/3, and thereby causes a conformational alteration [44]. Given that the binding site of cortactin partially overlaps with WASP [45], WASP-cortactin-Arp2/3 complex may be inform of a ternary complex [46]. Otherwise, cortactin can take the place of WASP to combine the Arp2/3 complex [30], and then stabilize branches and liberate WASP for a next new cycle [46]. An alternative mechanism includes cortactin binding to WASP by the SH3 domain [28]. The SH3 domain may enhance actin nucleation via binding to the WASP, WIP, and dynamin 2 [46]. Once the Arp2/3 is activated, it will bind to a pre-existing actin filament and then produce a new filament, which with a 70° branch [47]. When the structure is formed, cortactin stabilizes branched actin networks. Inhibition to debranching appears to be a unparallel and essential function of cortactin [48].

**Figure 2:**
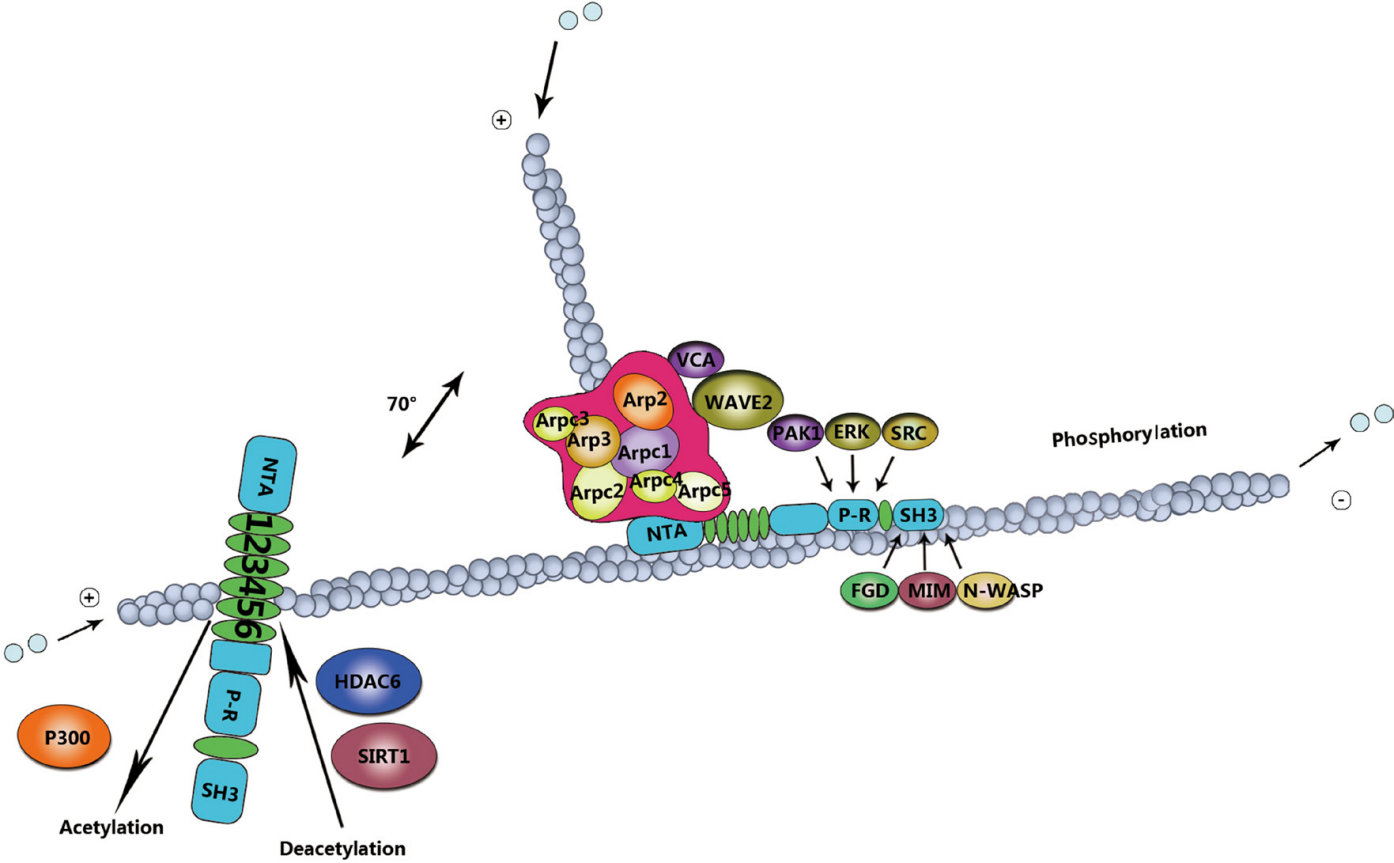
Mechanism of cortactin post-translation modifications in cell migration The role of cortactin in actin assembly depends on phosphorylation and acetylation events. Erk phosphorylation regulates the intramolecular interaction or induces conformational changes in cortactin, leading to binding to the N-WASP-Arp2/3 complex for actin polymerization. Src activation can directly nullify the Erk effect, ensuring actin disassembly. This intricate balance in actin organization is important for cell migration, acetylation by p300, and deacetylation by HDAC6 and SIRT1.

Several studies have shown that activation of N-WASP is induced by cortactin involved in binding to its proline-rich domain and decreasing auto-inhibition [19, 49]. Furthermore, the SH3 domain contains many adaptor proteins, for instance, Nck and growth factor receptor-bound protein 2 (GRB2) [50]. These adaptor proteins have ability to stimulate N-WASP by binding to the proline-rich domain [51, 52]. In a word, the SH3 domain has capability to stimulat the Arp2/3-mediated actin polymerization by means of activating N-WASP, and thereby promotes cell migration [53]. In addition, Src-mediated tyrosine phosphorylation of cortactin regulates cell movement (Figure 2).

## CORTACTIN PROMOTES CELL MIGRATION

### The role of cortactin in podosome and invadopodia formation

Cell migration through interstitial tissues is related to specialized characteristics of the migrating cell, in particular the development of proteolytic membranous invadopodia and podosome [54]. Invadopodia are actin-based membrane protrusions which are localized on the basal surface of invading cells, and these cells involved in a serious of cellular processes, for instance the assembly of branched actin, adhesion, cell signaling and proteases secretion that spatially converge to promote ECM remodeling [53, 55, 56]. Podosomes are similar to invadopodia, whereas the former was found in normal cells which play a role in crossing tissue barriers or ECM remodeling [57].

Cortactin are capable of stimulating the endurance of invadopodia [58], except, inducing formation of the protrusions [58–62]. Cortactin also improve the capability of the cells to produce dominant invadopodia in response to stimulation of growth factor [61]. Furthermore, cortactin facilitates the novel adhesions formation at the edge of cells, which may contribute to invadopodia persistence and migration [58]. Defects in invadopodia endurance, adhesion molecule, and cellular move ability can be fully supplemented via the modify function of the cortactin derivatives which only include the Arp2/3 and F-actin binding domain [63, 64]. This suggests that cortactin is responsible for direct stimulation of the assembly and/or stability of branched actin mediated by Arp2/3 [32, 58]. The cortactin phosphorylation via Src kinase is required for podosome formation mediated by cortactin [17, 65]. Webb et al. demonstrated that cortactin is crucial for the formation of podosome, an intact SH3 domain which is necessary for cortactins transposition to early podosomes [17, 66]. The SH3 domain stimulates N-WASP, thereby suggesting that cortactin are likely to furnish a scaffold for the polymerization of actin [17, 67]. On the flip side, the actin-binding repeated domain of cortactin was founded playing a role in the advanced stages of podosome formation [17, 66]. As above mentioned, the dynamin is necessary for the formation of podosome in normal cells [17, 68, 69]. The interaction between cortactin and WIP may be involved in the formation of podosome, suggesting that when both the proteins consist in the podosomes, the co-expression of them will promotes the membrane protrusions [17, 35].

### Post-translational modifications of cortactin in cell migration

#### Tyrosine phosphorylation of cortactin

Cortactin interacts with numerous binding partners [24], and is activated by several post-translational modifications. Cortactin was initially characterized via its tyrosine phosphorylation activity, in v-Src transformed cells [1, 47]. Src kinase phosphorylates cortactin at three tyrosine residues, include Y421, Y466, and Y482 [17, 47]. Tyrosine phosphorylation occurs primarily at Y421 and Y466, the two sites are localized within the proline-rich domain [70, 71]. However, mapping of post-translational modifications by mass spectrometry has resulted in the identification of additional targets, including Y482 [72]. Interestingly, tyrosine phosphorylation of cortactin occurs with a progressive manner, with the phosphorylation initially occurring at the site of Y421 and whereafter, at the site of Y466 [17, 73]. The phosphorylation of Y421 via Src creates a binding site for the cortactin SH2 domain [74–76] that is whereafter phosphorylation of Y466, or other sites. Several non-receptor tyrosine kinases have been involved in the phosphorylation of cortactin [77], including members of Src kinase family, ABL kinase family, FER, and Syk [78–80]. It has been demonstrated that in cancer cells, activation of hepatocyte growth factor receptor (HGFR, also known as MET), human epidermal growth factor receptor 2 (HER2, also known as ERBB2), and tyrosine kinases stimulates cortactin phosphorylation [81].

At the mechanistic level, tyrosine phosphorylation triggers the recruitment of SH2-domain proteins, including kinases, and the cytoplasmic protein Nck adaptor protein 1 (Nck1), which links cortactin with N-WASP and WIP, enhancing the activation of the Arp2/3 complex [3, 4, 82]. These findings are consistent with the observation that tyrosine phosphorylation of cortactin induces lamellipodia protrusion and cell migration [83]. The phosphorylation of tyrosine involved in cortactin functionality is a intricate phenomenon, unmasked a mass of processus biologique involving cortactin [17]. So the level of cortactin phosphorylation is positively related to its capability to lead to cell migration [17, 84].

#### Serine/threonine phosphorylation of cortactin

In addition to tyrosine phosphorylation, cortactin can be phosphorylated by serine/threonine kinases, including extracellular signal-regulated kinase (ERK)/MAP kinases, protein kinase D (PKD), and p21-activated kinase (PAK) [85]. Indeed, ERKs, also known as MAPKs can phosphorylate cortactin at residues S405 and S418 [19]. The serine phosphorylation mediated via ERK may promote the process of the SH3 domain interacts with sequences within the proline-rich domain. The interaction impels cortactin to bind N-WASP, increasing the polymerization of actin mediated via the Arp2/3 [7]. PKD phosphorylates cortactin at S298, which increases cell migration due to the activation of the Arp2/3 [5, 86]. Besides the S298, PKD also phosphorylates S348 in invadopodia of breast cancer cells [87]. Reaction to the growth factor receptor stimulation [88], the serine/threonine-protein kinase PAK3 phosphorylates cortactin at S113 [85]. PAK1 phosphorylates cortactin at S405 and S418, downstream of RAC1 and CDC42 [77]. Both the RAC1 and CDC42 are components of the small GTPases Rho family that are required for localizing cortactin at the cellular edge, increase the association of cortactin with N-WASP [89, 90].

#### Acetylation of cortactin

In addition to phosphorylation, cortactin activity can also be modulated by acetylation by histone acetyltransferase p300, and deacetylation by histone deacetylase 6(HDAC6) and sirtuin-1 (SIRT1) [91, 92]. Acetylation neutralizes charged lysine (Lys) residues within the binding domain of F-actin [93, 94], thereby abrogating the capability of cortactin to bind F-actin and decreasing cells motility [94, 95]. Recently, HDAC6 has been acting on enhance the binding capacity of cortactin to actin filaments via modifying a charge patch in the repeats domain [95, 96]. Similar as in many cortactin regulatory mechanisms, acetylation appears to play an essential role in the cancer progression [97]. For example, HDAC6 expression is necessary for the the formation of invadopodia and degradation of ECM in breast cancer cells [97–100].

## THE ROLE OF CORTACTIN IN TUMOR INVASION

### Cortactin function in membrane trafficking and secretion of matrix metalloproteinases

Tumor cell metastasis progress depends on the activation of invadopodia for the degradation of ECM barriers [101]. Cortactin promotes membrane trafficking and recruits ECM-degrading proteinases to invadopodia sites, thereby cortactin made an essential role in the process of degradation of ECM associated with invadopodia [102]. Membrane trafficking plays a role in the formation of invadopodia, for this function is mainly dependent on the secretion of protease in the process of ECM degradation [103, 104]. One possible way to explain this mechanism is promote cell migration and invasion via the affection of cortactin on the proteins, including actin polymerization and/or bridging membrane trafficking to the actin cytoskeleton to increase membrane trafficking. In this process cortactin represents an indirect link between special transmembrane receptors and the actin cytoskeleton. This usually happens through the interaction of the SH3 domains with sub-membranous scaffolding proteins.

**Figure 3:**
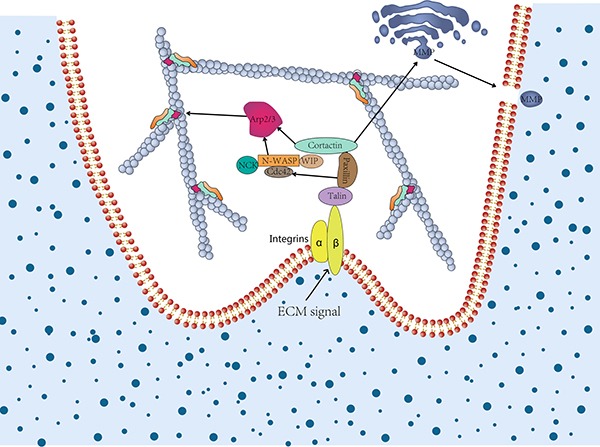
The role of cortactin in invadopodia formation and extracellular matrix degradation In cancer cell invasion, cortactin contributes to invadopodia formation and extracellular matrix (ECM) degradation. The former relies on the activation of N-WASP via Nck, Cdc42 via paxillin, and coactivation of the Arp2/3 complex. The latter relies on secretion of MMPs and vesicular trafficking to invadopodia via regulation of post-Golgi trafficking or vesicle capture. Once ECM degradation is established, invadopodia may live longer due to positive feedback.

Cortactin and its several binding proteins are involved in the process of protein trafficking (Table 1). It has been reported that cortactin recruitment preceded the proteases trafficking to the sites of ECM degradation [105]. In addition, the cortactin and phosphotyrosine signaling may be essential to delivery and/or activate the proteases which associated with invadopodia [106]. In addition, cortactin has been shown to be crucial for trafficking the key invadopodia proteases, including matrix metalloproteinase 1 (MMP1), MMP2, and MMP9 from late endosomes to the plasma membrane, which play a crucial role in the invasive potential of cancer cells (Figure 3) [53, 107, 108]. Recent research conducted by Zhao et al. showed that a synergistic reaction was exhibited on cortactin and Exo70, which could simultaneously stimulate the secretion of MMPs and thus regulate tumor invasion [109]. Exo70 a key subunit of the exocyst complex, was involved in regulating the cell migration and invasion through interaction with microfilaments [38].

### Cortactin is crucial for tumor invasion

Cortactin have essential role in human neoplasia [8]. A large number of evidences indicate that cortactin is overexpressed in various types of human cancers [47], such as head, neck, and esophageal squamous carcinomas, colorectal carcinoma, gastric cancer, hepatocellular cancer, breast cancer, and ovarian cancers [9, 110–113]. Cortactin overexpression is frequently due to chromosomal amplification of the 11q13 region [111] that is localized in the cortactin-encoding EMS1 gene [114]. This gene has a large region containing several known or potential oncogenes [53]. This amplicon included several genes found in human tumors frequently, such as oncogenes Cyclin D1 (encoded by CCND1/PRAD1), INT2/Fibroblast growth factor 3 (FGF3), human serum thymus factor/FGF4 and BCL1 [24]. CCND1, a gene located in the chromosomal region. There are four ‘cores’ of gene amplification within the 11q13 regions, among which CCND1 and EMS1 have been identified as the prime candidate genes to promote their respective cores amplification [53]. Unlike many other genes, these cores are usually overexpressed upon amplification [24]. Cortactin has been associated with poor prognosis estrogen receptor-negative breast cancer, whereas cyclin D1 has been associated with the positive [115]. The fact that the cortactin can still be amplified without cyclin D1 amplification [115], suggests that EMS1 amplification directly involved in tumor progression. Cell lines isolated from patients with 11q13 amplification exhibited increased levels of cortactin mRNA and protein, providing strong circumstantial evidence that cortactin overexpression plays an important role in tumors with 11q13 amplification. In addition, cortactin overexpression inhibited the degradation of epidermal growth factor receptor, leading to increased pro-mitotic receptor signaling [116, 117].

Recent studies have supported the cortactin role in metastasis, which is consistent with its function in cell motility and ECM degradation [118–120]. Indeed, cortactin expression is a key factor in ECM secretion, which dictates cell migration and may explain diverging theories regarding the importance of cortactin in cell motility [102]. For example, studies have shown that the loss of cortactin does not affect cell migration when cells grow on an ECM [121]. However, in the absence of an ECM, cells *in vitro* showed a pronounced defect in cell migration, suggesting that cortactin is crucial for cell migration in absence of an ECM [102].

## PERSPECTIVES

We reviewed the state of the art of researches that a Src kinase substrate, cortactin plays an essential role in regulating a myriad of cellular processes, such as actin polymerization, podosome/invadopodia formation, and tumor metastasis [88]. These cellular processes may be relevant to the etiopathogenesis of human cancer [17]. We reviewed the work done to identify the actin nucleation-promoting factor cortactin as a essential regulator of actin cytoskeletal dynamics. Cortactin a enables coupling of two key biological characteristics, and it can also regulating the nucleation and stabilization of branched actin networks mediated via Arp2/3 [29], for another its SH3 domain-mediated targeting to specific protein complexes. However, more insight is required to clear whether the multiple mechanisms are operative in different cellular functions, such as the cortactin as a scaffolding protein, stimulates the Arp2/3 directly or not, and the role in stabilization of branches [122]. We reviewed reports that the post-translational modifications of cortactin are essential in the process of cell motility and morphological changes. However, the results of phosphorylation remain controversial. For example, in invadopodia, the disruption of tyrosine phosphorylation sites of cortactin prevented cell edge protrusion, cancer cell invasion, and actin polymerization [82, 123]. However, other studies have shown that, disrupted phosphorylation promoted interactions between cortactin and N-WASP, and enhanced the stimulation of Arp2/3 [7].

We also reviewed the literature that the role of cortactin in tumor invasion. Cortactin affects cell aggressiveness via regulating MMP secretion and ECM degradation [102]. Known as an important regulator in ECM degradation, cortactin which is associated with invadopodia promotes membrane trafficking and recruits ECM-degrading proteinases to invadopodia sites. Recently, a report showed that a synergistic reaction exhibited on cortactin and Exo70, which could mediate the secretion of MMPs, and thus regulate tumor invasion [109]. However, the interaction mechanism between cortactin and Exo70 is unclear. Finally, we reviewed reports that cortactin is overexpressed in many cancers through 11q13 amplification. However, further studies are required to make clear the mechanization of the chromosome 11q13 amplification and cortactin overexpression in cancer cells.

In summary, cortactin is crucial for many cancer cellular processes. However, more insight is required to clarify the functions of cortactin in tumor progress and the tumor microenvironment, the binding domains of cortactin in promoting the aggressiveness of cancer cells. An intensive study of the cortactin role in tumor may lead to identifying some new therapeutic strategies to treat cancer.

## Abbreviations

Arp2/3 complex: actin related protein 2/3 complex
ECM: extracellular matrix
NTA: N-terminal acidic
SH3: Src homology
VCA: verprolin-cofilin-acidic
N-WASP: Wiskott-Aldrich syndrome protein
WIP: WASP-interacting protein
HGFR: hepatocyte growth factor receptor
HER2: human epidermal growth factor receptor 2
ERK: extracellular signal-regulated kinase
PKD: Protein kinase D
PAK: p21-activated kinase
HDAC6: deacetylase 6
SIRT1: sirtuin-1
MMP: matrix metalloproteinase
CCND1/PRAD1: cyclin D1
FGF3: fibroblast growth factor 3.
